# Radiation-Induced Chromosomal Aberrations and Immunotherapy: Micronuclei, Cytosolic DNA, and Interferon-Production Pathway

**DOI:** 10.3389/fonc.2018.00192

**Published:** 2018-05-29

**Authors:** Marco Durante, Silvia C. Formenti

**Affiliations:** ^1^Trento Institute for Fundamental and Applied Physics (TIFPA), National Institute for Nuclear Physics (INFN), University of Trento, Trento, Italy; ^2^Department of Radiation Oncology, Weill Cornell Medical College, New York, NY, United States

**Keywords:** radioimmunotherapy, chromosome aberrations, micronuclei, cyclic GMP–AMP synthase, stimulator of interferon genes, particle therapy

## Abstract

Radiation-induced chromosomal aberrations represent an early marker of late effects, including cell killing and transformation. The measurement of cytogenetic damage in tissues, generally in blood lymphocytes, from patients treated with radiotherapy has been studied for many years to predict individual sensitivity and late morbidity. Acentric fragments are lost during mitosis and create micronuclei (MN), which are well correlated to cell killing. Immunotherapy is rapidly becoming a most promising new strategy for metastatic tumors, and combination with radiotherapy is explored in several pre-clinical studies and clinical trials. Recent evidence has shown that the presence of cytosolic DNA activates immune response *via* the cyclic GMP–AMP synthase/stimulator of interferon genes pathway, which induces type I interferon transcription. Cytosolic DNA can be found after exposure to ionizing radiation either as MN or as small fragments leaking through nuclear envelope ruptures. The study of the dependence of cytosolic DNA and MN on dose and radiation quality can guide the optimal combination of radiotherapy and immunotherapy. The role of densely ionizing charged particles is under active investigation to define their impact on the activation of the interferon pathway.

## Introduction

The analysis of chromosome aberrations (CA) has been one of the first tools to study the mechanisms of radiation action in living cells. Being relatively easy to observe and measure, early cytological investigations focused on asymmetrical rearrangements observed at the first mitosis after irradiation. The classical theory of radiation action, developed by Douglas Lea almost a century ago (1), is largely based on CA data, and its basic principles are considered still valid today. With the discovery of fluorescence *in situ* hybridization (FISH), radiation cytogenetics entered in the “color” era (2) and many more details on the formation of radiation-induced CA could be discovered, especially those involving symmetrical-type rearrangements, complex-type exchanges, and intrachromosomal exchanges. Studies on molecular pathways of DNA damage response have largely increased our understanding of the link between the initial DNA lesions, especially double-strand breaks (DSB), and the formation of CA (3).

Chromosome aberrations can be easily assessed in peripheral blood lymphocytes (PBL), stimulated to grow *ex vivo*, and for this reason they are a useful tool for radiation biodosimetry (4). It is indeed quite easy to get blood samples both from radiation workers (in case of a nuclear accident) or from patients treated with radiotherapy. The MN assay can also be performed in automatic or semi-automatic image analysis system (5, 6), making possible the scoring of many thousands of cells in short time. For many years, dicentrics and MN measurements have been used for biodosimetry. With FISH-painting, it became possible to look at stable CA and, therefore, to perform retrospective biodosimetry. Inter-individual variability in measurements of radiation-induced CA in PBL (7) has triggered many efforts to investigate *ex vivo* measurements as predictive assays of individual susceptibility to radiation therapy. Despite these high expectations, the results have been disappointing, since no clear correlation with individual toxicity could be established (8, 9). CA in PBL are also considered an early biomarker of late cancer risk. However, even if this observation is supported by molecular epidemiology studies (10), it only applies to large population studies and has little application to individual prediction of risk. Therefore, with the recent onset of precision medicine based on genome sequencing in radiotherapy (11, 12), CA studies became less appealing and somehow regarded as “old style” compared to the modern genome-wide association studies and epigenetic techniques.

Recently, the extensive experience from radiation-induced CA studies has been re-visited in a completely different context: immunotherapy. Immunotherapy is nowadays generally acknowledged as a most promising strategy for cancer treatment, including the setting of metastatic disease (13, 14). Because of the pivotal trial in metastatic melanoma, innumerable clinical trials are testing immune checkpoint blockade agents in multiple solid tumors. In locally advanced and metastatic solid tumors, immunotherapy is often combined with a local treatment (15). The combination of radiotherapy and immunotherapy (radioimmunotherapy) is particularly promising, because radiation elicits immune response pathways that can boost the action of drugs that either stimulate the immune system or block immune suppressive signals (16–22).

## DNA Damage and Innate Immunity

The molecular signaling linking the initial radiation effects (DNA damage and CA) to immune stimulation has been recently discovered in studies of autoimmune diseases (23). The cyclic GMP–AMP synthase (cGAS) is a cytosolic DNA sensor that, upon binding double-stranded DNA (dsDNA), activates the stimulator of interferon genes (STING) endoplasmatic reticulum adaptor protein (24, 25). STING induces type I interferon and other cytokines, key mediators of BAFT-3 dendritic cells recruitment for cross presentation and immune response (26, 27), and it is, therefore, important for successful cancer immunotherapy (28, 29). In murine models STING activation in the tumor microenvironment and/or in the cancer cells enhances radiation-induced antitumor immunity (30, 31) and can prevent radiation-induced acute intestinal tissue injury (32). It has to be noted, however, that the role of the STING activation in immune response can be multi-faceted. Radiation-induced STING activation may be immunosuppressive due to myeloid-derived suppressor cell infiltration (33), and may promote invasion and metastasis by upregulating the NF-κB pathway (34). Notwithstanding the complexity of the pathways and the possibility of opposite effects, cytosolic sensors of nucleic acids are extensively studied as targets for immunotherapy (35).

But how can dsDNA leak into the cytoplasm? The mechanism may actually be the one well known from radiation-induced CA studies: formation of micronuclei (MN).

## Micronuclei

**Figure 1:**
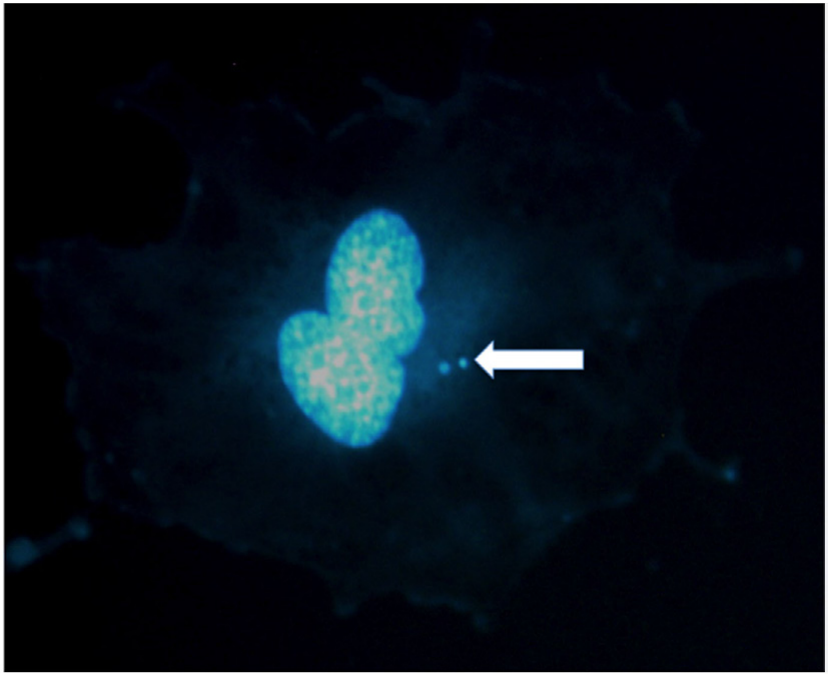
Micronuclei in the cytoplasm of a human umbilical vein endothelial cell exposed to X-rays. Two micronuclei are visible in this binucleated cell stained in DAPI. Photo courtesy of Dr. Alexander Helm.

**Figure 2:**
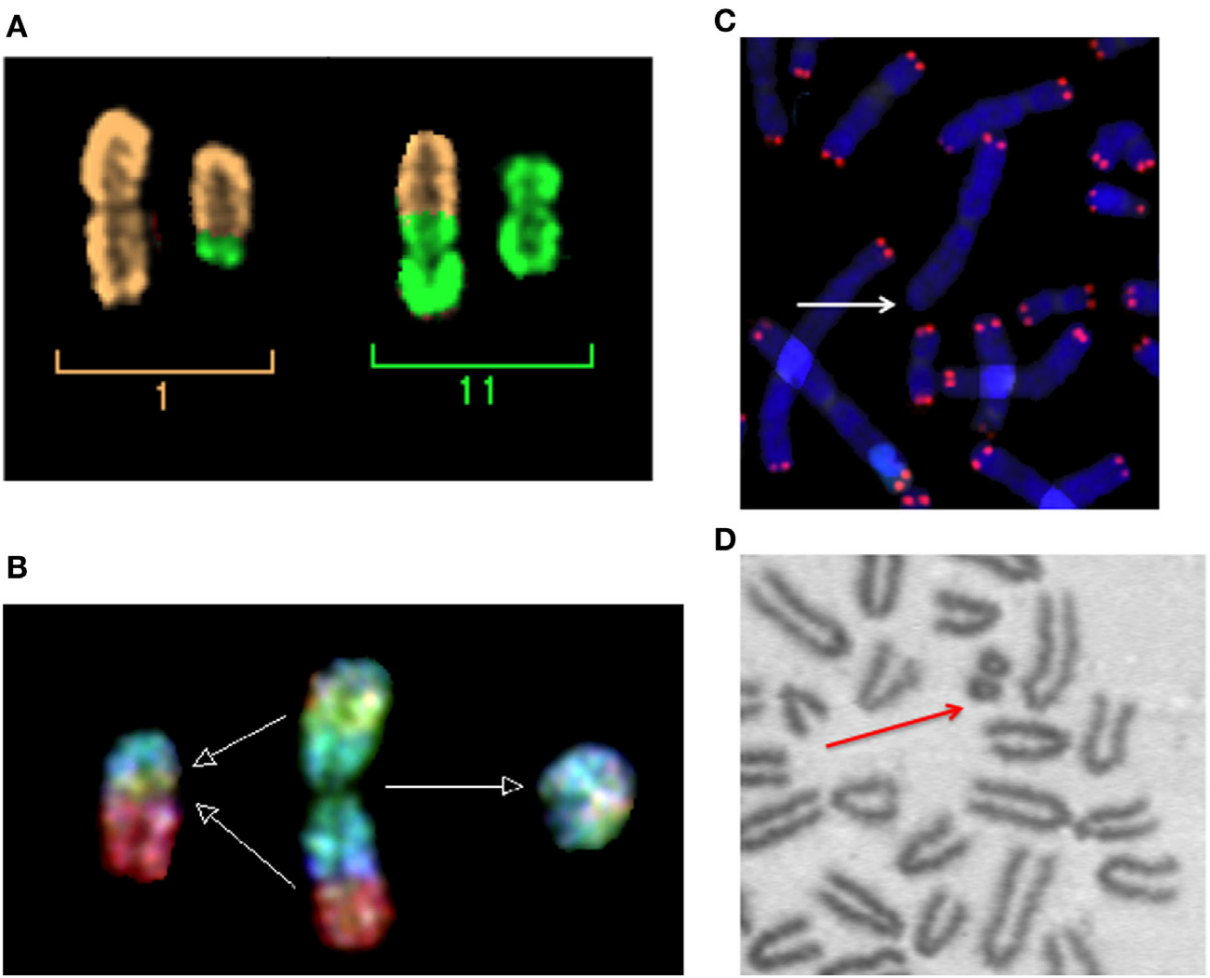
Micronuclei originate from radiation-induced chromosome aberrations. Acentric fragments in irradiated cells are shown in this panel. **(A)** A dicentric chromosome and associated acentric fragment between chromosome 1 and 11 in a human peripheral blood lymphocytes exposed to heavy ions. Image visualized by mFISH. **(B)** Formation of a radiation-induced ring and its associated acentric fragment visualized by R × FISH (cross-species color banding). A normal chromosome 3 is shown in the middle, the centric ring on the right, and the acentric fragment resulting from the joining of the two residual fragments in the two different arms on the left. **(C)** A terminal deletion induced by heavy ions in human lymphocytes. The lack of telomere signal indicates that a fragment has been lost. **(D)** An interstitial deletion at the first mitosis following exposure of G0-phase mouse fibroblasts to X-rays. Interstitial deletions appear often as double minutes. In this photomicrograph of Giemsa-stained mitotic chromosomes, it is clear their nature of acentric rings resulting from the asymmetrical intra-arm intrachange.

Micronuclei are small nuclei found in the cytoplasm of mammalian cells (Figure 1). They can originate either by acentric fragments or whole chromosome loss at anaphase (36). Spontaneous MN are found at low level in different normal human tissues and cells, and are produced by genotoxic stress and exposure to clastogenic or aneugenic agents. MN are generally scored in cytochalasin-blocked binucleated (BN) cells. With the cytochalasin-B treatment, MN can be scored in cells attempting their first mitosis (37). The two nuclei of the blocked cell can be joined by a nucleoplasmic bridge (NPB) in the presence of dicentrics or polycentric aberrations (38). Following exposure to ionizing radiation, CA are formed including acentric fragments (Figure 2) originating from asymmetrical intra-arm (interstitial deletions) or inter-arm (centric rings) intrachanges, asymmetrical interchanges (dicentrics), terminal deletions, or incomplete exchanges (39). Using conventional, solid staining in Giemsa, observed interstitial deletions in human PBL at the first mitosis after radiation exposure are about 60% of the dicentrics (40). However, fragments from rings or dicentrics are rather large, while many interstitial deletions are small, often below the detection limit of many cytogenetic techniques (41, 42). The recent method of directional genomic hybridization has uncovered a large number of small inversions (42, 43), the symmetrical counterpart of interstitial deletions. It is also known that ionizing radiation induce chromosome missegregation and, therefore, aneuploidy (44, 45). Micronuclei containing whole chromosomes can be easily detected using FISH with centromeric DNA probes or antikinetochore-antibody (CREST) probes. While the baseline frequency of CREST-positive MN is rather high (about 40% of the total background MN), the vast majority of radiation-induced MN are CREST-negative (46), suggesting that the main mechanism for the formation of radiation-induced MN is the formation of asymmetrical-type CA by DNA DSB misrepair.

## Micronuclei and Cytosolic DNA

If the damaged cell go through mitosis, MN can be transmitted to the daughter cells and thus persist much longer (47), especially in cells with mutated or lost p53 (48), as most cancer cells. Defective and delayed DNA replication occurs within MN, with impaired checkpoint arrest, leading to pulverization of the incompletely replicated MN DNA in the ensuing mitosis (49). This mechanism may explain the generation of chromothripsis (literally “chromosome shattering”), a single catastrophic event leading to multiple, up to thousands, chromosomal rearrangements (50–53).

Cytosolic DNA in MN can explain the activation of the cGAS/STING pathway following exposure to clastogenic agents and, specifically, ionizing radiation. In fact, relocalization of cGAS to MN following mitotic progression triggers inflammatory signaling (54) and interferon-stimulated gene expression is activated in cells containing MN (55). cGAS can sense the dsDNA in the cytosol because the MN nuclear envelope (NE) often collapses during interphase in cancer cells, due to defects in nuclear lamina assembly (56). Recently, using several human and murine carcinoma cells, it has been shown that the concentration of cytosolic dsDNA increases with radiation dose up to about 15–18 Gy, but then decrease at higher doses, probably because it is degraded by the activation of the DNA exonuclease Trex1 (31). Finding the correct dose for induction of sufficient dsDNA and immune-stimulatory signals *via* cGAS/STING pathway versus Trex1 activation (57) may be a key step to select optimal radiotherapy protocols during immunotherapy. Insights can be derived from the analysis of radiation dose- and quality-dependence of MN induction.

## Radiation-Induced Micronuclei

**Figure 3:**
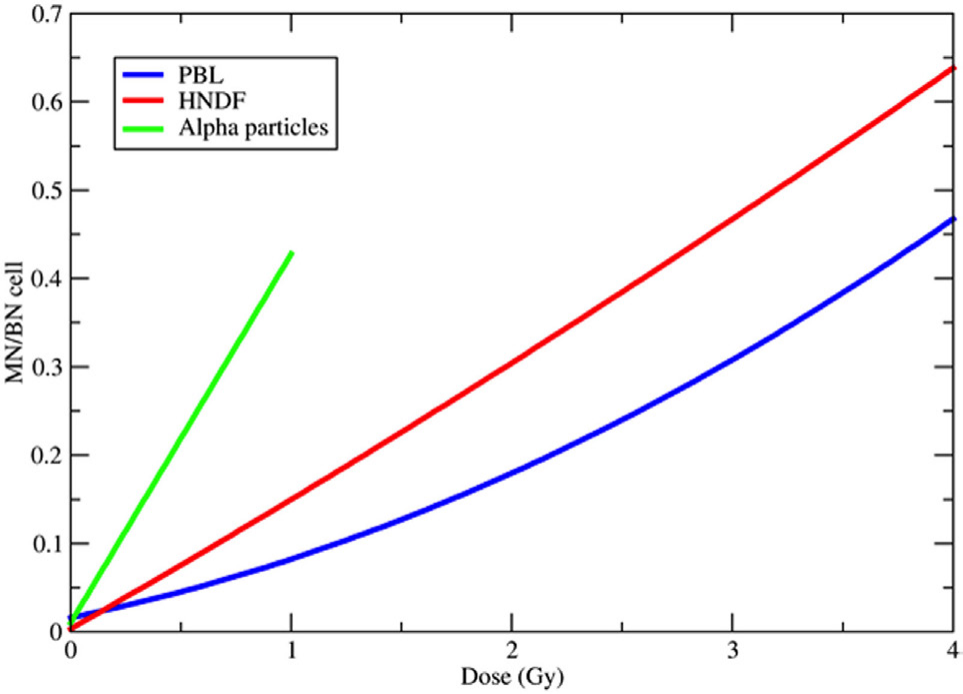
Examples of dose-response curves for the induction of micronuclei (MN) per binucleated (BN) cell. The blue curve is the weighted mean of the calibration curves used in 10 different European laboratories for MN biodosimetry using human peripheral blood lymphocytes (PBL) within the RENEB project (78). The equation is *Y* = 0.016 + 0.0508·*D* + 0.0155·*D*^2^, where *Y* is the frequency of MN per BN cell and *D* the ^60^Co γ-ray dose in gray. The red curve refers to human neonatal dermal fibroblasts (HNDF) exposed to ^60^Co γ-ray (59). The equation used by the authors to fit their data was *Y* = 0.033 + 0.1423·*D* + 0.0041·*D*^2^. The green line refers to human PBL exposed to ^239^Pu α-particles (61). Data points (all at doses <1 Gy) were fitted by the function *Y* = 0.098 + 0.418·*D*.

**Figure 4:**
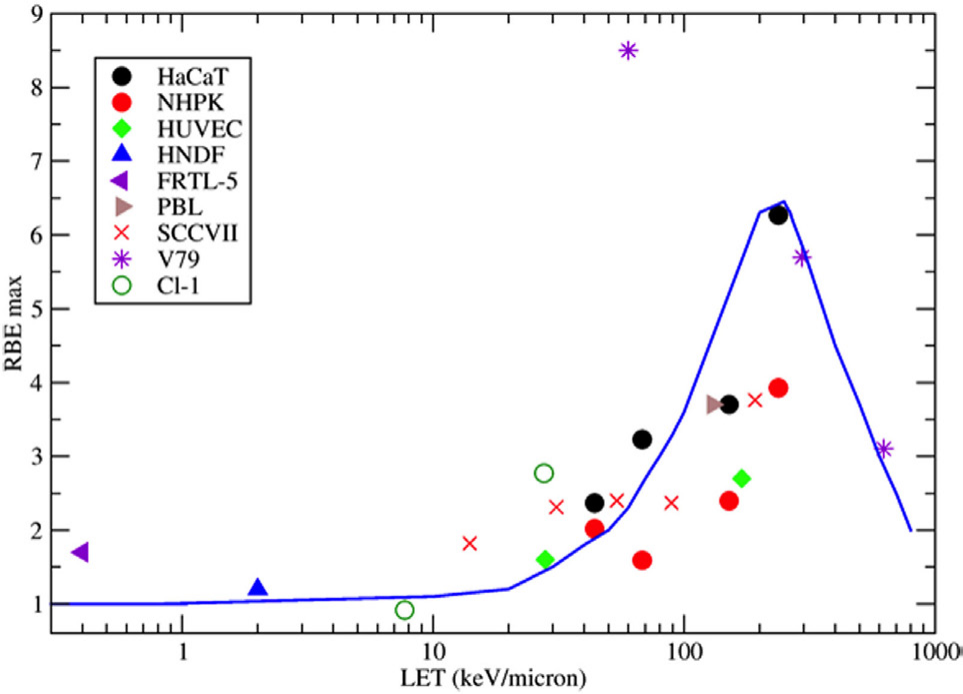
Collection of measured relative biological effectiveness (RBE) data for the induction of MN per BN cell after exposure to protons, α-particles, or energetic heavy ions. RBE was calculated as the ratio of the initial slope of the dose-response curves for ions and photons as measured by the same authors. Different symbols refer to different cell types. HaCaT = spontaneously immortalized adult human keratinocytes (63); NHPK = normal human neonatal epidermal keratinocytes (63); HUVEC = human umbilical vein endothelial cells (67); HNDF = human neonatal dermal fibroblasts (59); FRTL-5 = Fischer rat thyroid cell line (60); PBL = human peripheral blood lymphocytes (61); SCCVIII = mouse squamous cell carcinoma (64); V79 =Chinese hamster fibroblasts (62); Cl-1 = Chinese hamster fibroblasts (46). The blue line is a guide for the eye.

Background yield of MN shows large variation among cell types and, in human PBL, significant inter-individual variability, ranging 0–40 per 1,000 BN cells (4). The dose-response curve in the range 0–4 Gy for the induction of MN/BN is generally considered linear-quadratic for low-linear energy transfer (LET) X- or γ-rays (36) and becomes linear for high-LET neutrons (58), protons (59, 60), α-particles (61), and heavy ions (62) (Figure 3). This behavior reflects the dose–response curve for the induction of CA (4). From these classical data, more dsDNA is expected to be produced by increasing dose. At the same dose, high-LET charged particles are more effective in the induction of MN (63–67) than sparsely ionizing X-rays. The relationship between relative biological effectiveness (RBE) and LET, based on the data available in the literature, is shown in Figure 4 and has the typical trend observed for other endpoints, such as CA (68), mutations (69), cells killing (70), or neoplastic transformation (71). Also consistent with the sparing effect observed at low dose rate for most cellular endpoints, MN yields are reduced for chronic compared to acute exposure (72, 73). MN have been often measured in PBL from individuals exposed to radiation because of nuclear accidents or for cancer therapy. In radiotherapy patients, the increase of MN in PBL is generally linearly correlated to the number of fractions (74–76) and strongly depends on the irradiated volume during the treatment (77).

**Figure 5:**
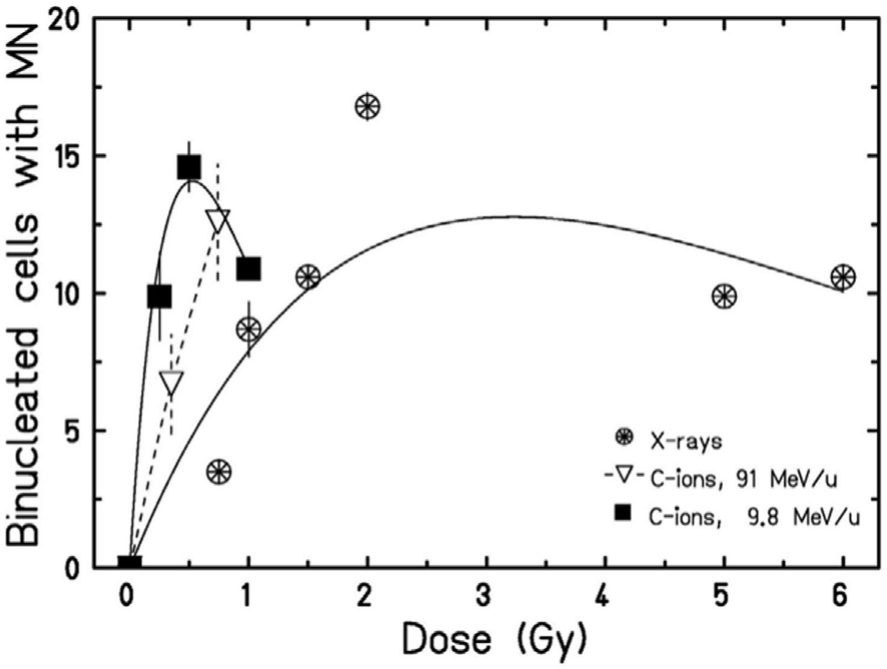
Dose-response curves for the induction of MN in HUVEC cells exposed to X-rays or accelerated carbon ions at two different energies. A curvature at high doses can be observed. Figure reproduced with permission from Ref. (67).

However, data in Figures 3 and 4 are relative to MN frequency per BN cell using the cytokinesis-block method. The time between irradiation, supplementation of cytochalasin-B, and harvest, are selected to block as many cells as possible reaching the first mitosis, and depends on the cell type. In PBL, cytochalasin-B is added 24 h after irradiation and cultures are fixed 72 h after irradiation with some differences among laboratories (78). The measured MN yield is normalized to the cytokinesis-blocked BN cells, i.e., those reaching mitosis. The problem is complicated by the cell-cycle delay and checkpoint block. Cells with less DNA damage tend to arrive in mitosis earlier than those heavily damaged (79), and the effect is stronger for densely ionizing (high-LET) radiation, where the dose distribution is non-uniform and the cell-cycle delays more pronounced (80, 81). Consistently, the yield of MN induced by α-particles or neutrons was indeed found to increase by increasing the harvest time from 72 to 120 h (82). This leads to a downward curvature in the dose-response curve when MN are measured at the same harvest time, and the decrease in BN cells carrying MN occurs at lower doses for high-LET compared to low-LET radiation (63, 67) (Figure 5).

## Fractionation and Radiation Quality in Immune Response

In the context of radioimmunotherapy, these data have an impact on the protocol to be used to optimize the immune response. A general trend in radiotherapy is the use of hypofractionation (83–85) and accelerated charged particles (86–88). In both cases, the combination with immunotherapy may be advantageous compared to conventional, fractionated X-ray therapy (89, 90). A few pre-clinical studies address the impact of the dose/fraction on the immune response (91–93). In a poorly immunogenic mouse tumor models not expressing model antigens, Dewan et al. (94) observed a strong abscopal response using anti-CTLA-4 antibody combined to fractionated (3 × 8 Gy) X-rays, while the effect was lost with a single dose of 20 Gy. Similarly, Schaue et al. (95) found that a medium size dose per fraction (7.5 Gy) was more effective than a single high dose of 15 Gy in eliciting immune response in a mouse melanoma model. Within the cGAS/STING pathway discussed above, the data on micronuclei seem to indicate a complex radiation dose- and quality-dependence. If MN elicit the proinflammatory immune response (96), the classical radiobiology data described above can now help to guide the radiotherapy schedule. Very high doses delivered in single fractions may reduce the yield of micronuclei (Figure 5), or greatly delay their appearance. Trex1 is involved in the resolution of NPB caused at mitosis by dicentric chromosomes (97), and is, therefore, activated at high doses. Trex1 activation at high doses removes available dsDNA from the cytosol, and will then inhibit the cGAS/STING activation (98). However, Vanpouille-Box et al. (31) found a threshold for the Trex-1 activation around 8–15 Gy, suggesting that quite large dose/fraction compared to the conventional 2 Gy/fraction, can be used in combined treatments. Defining the right balance between the inductions of MN/dsDNA without triggering the suppressive mechanisms elicited at high doses appears to be specific for each tumor type (93). In fractionated radiotherapy, cancer cells containing MN can be exposed and, because of the defective DNA repair mechanism in the MN (50), the MN DNA will be cut in smaller fragments. This can also affect the activation of the STING–interferon pathway, because cGAS catalytic activity depends on the dsDNA size (99, 100).

Similar arguments apply to fractionation with particle therapy. Charged particles are more effective in the induction of MN (Figure 4) but also induce more severe mitotic delays than X-rays. Fractionation reduces the yield of MN induced by X-rays compared to single fractions, but the sparing effect is reduced for high-LET radiation, and in fact no difference in MN frequency was observed in PBL exposed to 3 Gy fast neutrons delivered in a single fraction or in two equal fractions (101).

## NE Ruptures

Can nuclear DNA fragments leak in the cytoplasm independently of the formation of MN? Small dsDNA is found in the cytoplasm after exposure of thyroid cells to electric pulses (102, 103). It is well known that densely ionizing radiation induces a high fraction of small DNA fragments (104–108), below the resolution of optical microscopy. The Ku-heterodimer, essential for the non-homologous end-joining (NHEJ) repair pathway, may fail binding fragments <40 bp (109) leaving them essentially free to drift in the cell. Supporting this hypothesis, the RBE of high-LET heavy ions is ~1 in Ku^−/−^ (NHEJ-deficient) cells (110), suggesting that the higher effectiveness of heavy ions in the induction of cell inactivation or CA is due to the inability of NHEJ to cope with very small fragments. While MN formation requires the cell to enter mitosis, the question is whether small DNA fragments can leak in the cytoplasm during interphase. This may be possible *via* transient NE rupture, a loss of nuclear integrity in interphase leading to mislocalization of nuclear and cytoplasmic proteins (111). NE rupture is enhanced by loss of p53 and Rb genes (112), and is, therefore, more common in cancer cells. Chromatin herniation and DNA DSB are found in the NE opening sites, which are rapidly repaired to prevent cell death (113). Interestingly, loss of the NE is observed during cancer cell migration and leads to cGAS accumulation at the rupture sites (114). Ionizing radiation promotes cell migration at sub-lethal doses (115–118). The linker of nucleoskeleton and cytoskeleton complex is responsible for both radiation-induced cell migration (119) and NE ruptures (120). Therefore, NE ruptures may become more likely following radiation exposure.

## Mitochondrial DNA

Cytosolic DNA is of course naturally present in mammalian cells, within the mitochondria. Mitochondria are the energy factories of the cells, and contain their own DNA (mtDNA), which encodes for some of the key genes in the electron transport chain. Cytosolic leakage of mtDNA also results in activation of the cGAS–STING pathway (121, 122). Can radiation-induced damage to mtDNA trigger the immune response? It is well known that mitochondria play an important role in radiation-induced effects. Mitochondria are sources of reactive oxygen species (ROS) and play a major role in the induction and persistence of oxidative stress following exposure to radiation (123, 124). They are also involved in non-targeted radiation effects (125, 126), suggesting their involvement in systemic responses such as post-radiation immunity. However, mtDNA is not a primary target of radiation. Recent track structure simulations suggest that the probability of DSB induction in mtDNA is very low at moderate doses, around 0.03% at 1 Gy for either γ-rays or densely ionizing radiation (127). The involvement of mitochondria in late radiation effects is more likely to be an indirect consequence of the ROS generation after irradiation and of the nucleus–mitochondria signaling pathway. Nevertheless, it is possible that mtDNA can leak in the cytosol following a direct hit from a charged particle. Using a particle microbeam, Walsh et al. (128) reported a rapid loss of membrane potential following focused irradiation of mitochondria with protons or C-ions. This result suggests that high energy deposition in the mitochondria can induce DSB in mtDNA and mitochondrial membrane damage. Even if depolarization was obtained only by focusing over 80 ions on the mitochondria (128), the results suggest that direct hits of heavy ions in the mitochondria may lead to leakage of mtDNA in the cytosol.

## Conclusion

**Figure 6:**
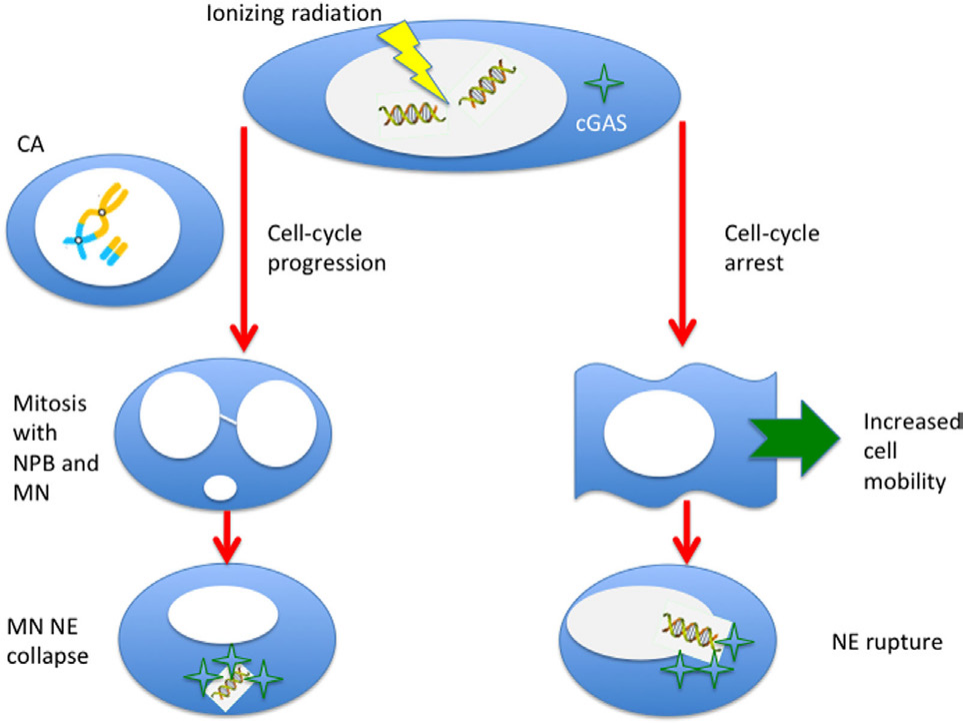
Ionizing radiation can induce cytosolic DNA, thus triggering the cyclic GMP–AMP synthase (cGAS)/stimulator of interferon genes (STING) pathway, in two different ways. If cells progress to mitosis carrying chromosome aberrations, MN (from acentric fragments) can be produced at the first mitosis along with nucleoplasmic bridges (NPB, from dicentrics). These micronuclei can be incorporated in the cytoplasm of the daughter cells and, following the collapse of the nuclear envelope (NE), can be sensed by the cGAS. Alternatively, even if the cell is blocked or delayed in the cell-cycle, radiation-induced DNA fragments can leak through a damaged NE. This effect can be more likely for very small fragments induced by densely ionizing radiation, and NE rupture can be triggered by the enhanced mobility of the cells following radiation exposure.

The link between immunotherapy and radiation-induced CA and MN is an example of “new tricks for old dogs.” First observed almost one century ago [see Ref. (1), chapter VI], they have been studied extensively mostly for biological dosimetry and as surrogate markers of cell death. The recently discovered role of cytoplasmic DNA sensing in immunity, derived from infectious disease studies, makes this experience now tremendously useful to understand the mechanism that underlie synergy of radiotherapy and immunotherapy in cancer treatment. Radiation is an S-phase-independent clastogen, and is a powerful inducer of MN for cells exposed in all cell-cycle phases. Moreover, radiation enhances cell mobility, and the cytoskeleton stress during motion can produce ruptures in the NE and additional leak of DNA fragments. Therefore, there seem to be at least two pathways for the production of cytosolic DNA by radiation (Figure 6): the first is dependent on cell-cycle progression and MN formation, the second on mobility and NE ruptures. A third possibility may arise from direct damage of mitochondria. The dose-response curve for MN induction is well characterized, and saturation at high doses by Trex1 activation suggests that fractionation may be more effective in eliciting STING–interferon pathway. The effect of repeated exposure to radiation of the MN DNA has not been sufficiently studied and, in this new context, seems to be important to characterize the effect of fractionation. Studies at high doses are also missing. Many experiments using densely ionizing radiation (neutrons, α-particles, heavy ions) have been performed, but a better characterization of cytosolic dsDNA, which can also come from very small fragments after high-LET exposure, is warranted. There are also no experiments addressing the activation of Trex1 and STING after exposure to protons or carbon ions, currently used in particle therapy. Radiation-induced CA and MN are likely to provide important contributions to modern molecular medicine in oncology.

## Author’s Note

The paper is the Inaugural Paper of MD as Editor of Frontiers in Oncology.

## Author Contributions

Both authors collected the literature and wrote the manuscript.

## Conflict of Interest Statement

The authors declare that the research was conducted in the absence of any commercial or financial relationships that could be construed as a potential conflict of interest. The reviewer HL and handling Editor declared their shared affiliation.
